# In-depth analysis for TKI-driven real-world management of 201 CML patients using TFR

**DOI:** 10.3389/fphar.2025.1673056

**Published:** 2025-11-06

**Authors:** Mihnea Lucian Micu, Simion Florin Cira, Mihnea Zdrenghea, Anca Bojan, Andrada Parvu, Tunde Torok-Vistai, Anca Vasilache, Laura Urian, Bancos Anamaria, Laura Ioana Jimbu, Maria-Elena Santa, Diana Lighezan, Adrian Bogdan Tigu, Andrei Ivancuta, Adrian Trifa, Elena-Cristina Selicean, Delia Dima, Rus Ioana Codruța, Ciprian Tomuleasa

**Affiliations:** 1 Department of Personalized Medicine and Rare Diseases - Medfuture Institute for Biomedical Research/Department of Hematology, Iuliu Hatieganu University of Medicine and Pharmacy, Cluj-Napoca, Romania; 2 Department of Hematology, Victor Babes University of Medicine and Pharmacy Timisoara, Timișoara, Romania

**Keywords:** bioinformatics & computational biology, machine learning, chronic myeloid leukemia, treatment free remission (TFR), real world evidence (RWE)

## Abstract

**Background:**

Tyrosine-kinase inhibitors (TKIs) have reshaped chronic myeloid leukemia (CML) outcomes, but real-world data from Eastern Europe remain scarce.

**Methods:**

We retrospectively analyzed 201 adult patients with CML managed at the Cluj-Napoca Department of Haematology (January 2001–December 2024). A semi-automated pipeline utilizing a Large Language Model was developed to extract structured data from unstructured medical discharge report text, with all patient identifiers removed to ensure anonymity. We captured demographics, disease phase, line-specific TKI use, adverse events (AEs), treatment-free remission (TFR) eligibility, TFR attempts, continuation, laboratory data and discontinuation. Machine learning models were trained to predict TFR potential.

**Results:**

Patients <60 years old, 101/201 (50.2%), and ≥60 years old, 100/201 (49.8%) were nearly equal in number. 53.7% were male. At diagnosis, 94.5% were in chronic phase. First-line treatment comprised imatinib in 108/201 (53.7%), dasatinib in 56/201 (27.9%), and nilotinib in 37/201 (18.4%). Second-line therapy (*n* = 64) was dominated by dasatinib (64.1%) and nilotinib (28.1%). Third- and later-line regimens increasingly incorporated bosutinib, ponatinib, and asciminib. Fourteen patients (7.0%) achieved sustained treatment-free remission (TFR). Among these, 3 had received imatinib, 3 dasatinib, and 8 nilotinib. An additional 31 patients (15.4%) were TFR-eligible but still on therapy—16 of them after imatinib, 9 after dasatinib and 6 after nilotinib. Imatinib achieved MR4+ in 29% of exposures and nilotinib in 43.3% of third-line uses, underscoring its role as the cohort’s most effective TFR-enabler. Predictive modelling for TFR potential using a Random Forest classifier achieved high accuracy (85.4%), with top predictors being whether a patient had ever achieved a deep molecular response (achieved_mr4_ever) and the best response after the first year (best_response_after_year1), highlighting the importance of both depth and timing of molecular remission. Adverse events led to discontinuation in 17/105 imatinib (16.1%), 23/105 dasatinib (21.9%), 9/68 nilotinib (13.2%), 6/13 bosutinib (46.2%), 4/11 ponatinib (36.4%), and 2/11 asciminib (18.2%) exposures. The most common imatinib discontinuation cause was loss of therapeutic response (34/105; 32.4%).

**Conclusion:**

In this Romanian center, imatinib was the predominant front-line TKI, reflecting both its earlier availability as the sole treatment option and the durable responses achieved by many long-term patients, but second-generation agents are increasingly used in over 40% of first-line starts. TFR uptake is limited despite a sizeable eligible population. Machine learning models demonstrate that both the depth and kinetics of molecular response are critical for predicting TFR potential. Prospective optimization of molecular monitoring and discontinuation protocols may broaden TFR success.

## Introduction

Chronic myeloid leukemia (CML) is a clonal myeloproliferative neoplasm that originates in a pluripotent hematopoietic stem cell and is biologically defined by the BCR-ABL1 oncoprotein. The advent and refinement of tyrosine-kinase inhibitors (TKIs) have transformed CML from a uniformly fatal leukemia into a chronic, highly controllable disease whose life expectancy now approaches that of the general population (Jabbour and Kantarjian, 2024). The pathognomonic lesion in CML is the Philadelphia chromosome, t(9; 22)(q34; q11), which juxtaposes BCR on chromosome 22 and ABL1 on chromosome 9. The resulting BCR-ABL1 fusion encodes a constitutively active tyrosine-kinase that drives leukemogenesis through unregulated proliferative and anti-apoptotic signaling cascades (Cross et al., 2023). SEER projections estimate 9,560 new CML cases and an incidence of ∼1.9 per 100,000 persons in the United States for 2025, with a 5-year relative survival of 70.4% (National Cancer Institute, 2025). Concurrently, population-based analyses show 10-year overall survival exceeding 80% in the contemporary TKI era, a marked improvement over the pre-TKI period (Sasaki et al., 2023). High-dose ionizing radiation is the only firmly established environmental risk factor for CML, conferring a dose-dependent increase in leukemogenic risk (Little et al., 2023). Conversely, large cohort and registry data continue to show no consistent association with tobacco exposure, and major cancer-advocacy bodies acknowledge that no other modifiable risk factor has yet been validated (American Cancer Society, 2024). CML follows a triphasic course—chronic, accelerated, and blast crisis—defined by increasing blast burden and progressive clinical instability. Approximately 90% of patients are referred to the clinical hematologist in the chronic phase, where TKI therapy is most effective; progression to advanced phases portends inferior outcomes (Shah et al., 2024).

Nearly half of CML cases are discovered incidentally on routine blood tests, yet many patients still report fatigue, night sweats, low-grade fever, weight loss, or exertional dyspnea. Splenomegaly—present in 30%–50%—remains the hallmark physical sign, often causing left-upper-quadrant discomfort or early satiety (Cross et al., 2023; Shah et al., 2024). Long-term outcome is now primarily determined by depth and kinetics of molecular response rather than by baseline demographics. Real-world evidence shows 5-year overall survival ≥80% and median life expectancy near normal for patients achieving early molecular milestones (Sasaki et al., 2023; Zenebe et al., 2023). Imatinib established the paradigm of ATP-competitive BCR-ABL1 inhibition, but second-generation TKIs (dasatinib, nilotinib, bosutinib) introduced greater potency and activity against many imatinib-resistant mutations. Ponatinib, a third-generation agent, retains activity against the gatekeeper T315I variant, albeit with vascular toxicity concerns. Most recently, asciminib, the first-in-class STAMP inhibitor that targets the ABL1 myristoyl pocket, demonstrated superior 48-week major molecular response versus investigator-selected TKIs in the ASC4FIRST phase 3 trial (Hochhaus et al., 2024), and received accelerated FDA approval in October 20249. Serial quantification of BCR-ABL1 transcripts underpins response assessment. Early molecular response (≤10% at 3 months), major molecular response (≤0.1%), and deep molecular responses (MR4–MR4.5) correlate with progressively lower risks of progression and death. Emerging data on transcript variant biology and digital PCR methodologies promise even greater sensitivity for residual disease detection (Cross et al., 2023; Zenebe et al., 2023).

Primary or acquired TKI resistance arises through BCR-ABL1 kinase-domain point mutations (notably T315I and compound mutants), BCR-ABL1 over-expression, activation of bypass signaling pathways, or pharmacokinetic obstacles such as efflux pump overactivity. Comprehensive genomic profiling and mutation-directed TKI sequencing are therefore integral to contemporary management strategies10. International guidelines recommend that treatment-free remission (TFR) be attempted only in chronic-phase patients with ≥5 years of uninterrupted TKI therapy and ≥2 years of sustained MR4 (or deeper) who have never experienced TKI resistance. Prospective series now report successful TFR in 40%–55% of appropriately selected patients, with rapid molecular relapse salvageable by TKI re-initiation (Shah et al., 2024; Bourne et al., 2024).

## Methods

### Study design and population

A single-centre, retrospective chart review included adults (≥18 years) with Philadelphia-positive CML who initiated care between 01 January 2001 and 31 December 2024. The initial dataset comprised 10,028 patient entries from electronic health records.

### Data processing and automated extraction

To overcome the challenges of unstructured data, we developed a semi-automated pipeline to process narrative clinical text.

1. Filtering and Cohort Refinement: Records were first filtered to include only those mentioning “BCR-ABL” in the discharge report text or diagnostic of discharge fields. A multi-step deduplication process was then applied to consolidate records for each unique patient, resulting in a final analytical cohort of 201 patients.
2. Data Extraction with a Large Language Model: We utilized the Large Language Model (LLM) to parse the unstructured discharge report text. A detailed prompt was engineered to guide the model in extracting key variables into a structured JSON format. All patient data were fully anonymized prior to processing. Personally identifiable information was removed, and each case was assigned a unique study identifier. The LLM received only anonymized discharge texts, with no access to patient identities. Linkage between extracted data and the original records was managed exclusively within the secure hospital data infrastructure, ensuring confidentiality and adherence to data protection regulations.
3. Manual validation of extracted data: All relevant data extracted for processing were manually verified by the author to ensure accuracy and maintain the integrity of the results. To further assess reliability, a validation procedure was performed. The initial dataset comprised 10,028 patient entries from electronic health records. After deduplication and removal of rows where one discharge was fully contained within another, the final dataset included 3,896 unique discharge summaries. Of these, approximately 1,000 discharges (about five per patient on average) were manually reviewed in full. Across the cohort, 19 out of 201 patients required intervention to correct details such as the succession of treatment lines and dates of therapeutic switches.
  These interventions were necessary at the patient level rather than the discharge level: extraction was accurate for individual discharge records, but inconsistencies showed up when compiling the complete treatment history, primarily because some early discharges did not document initial therapy lines that were later referenced in subsequent records. This outcome was expected, as the medical discharge summaries used as input to the LLM contained a median of only 1,054 words, while laboratory and blood work data were consistently written in machine-readable key–value pairs (e.g., HGB = 14.3 g/dL). As a result, all individual data points extracted—including BCR-ABL1 values, hemoglobin, leukocytes, thrombocytes, blasts, and their corresponding dates—were found to be perfectly accurate in all reviewed cases. This confirms that the extraction process was highly reliable, with only minor and explainable discrepancies identified at the aggregated patient level.
4. Structuring and Feature Engineering: The structured JSON output was parsed and flattened into analytical tables. This process created dedicated tables for laboratory data, BCR-ABL results, bone marrow aspirates reports, and blood smear analyses. Further feature engineering was performed to calculate variables such as patient age at diagnosis and the duration of each line of therapy.
5. Data Cleaning, Standardization, and Imputation: The structured data underwent rigorous cleaning. Treatment names and clinical phases were standardized (e.g., “Glivec” to “imatinib”). Missing longitudinal data, such as lines of therapy, were imputed using forward-(up to date of the next TKI) and backward-fill methods within each patient’s timeline manually

### Definitions

• Disease phases (chronic phase, accelerated phase, blast crisis) were defined according to the criteria applied by treating physicians at the time of care. Before 2013, phase classification reflected the prevailing diagnostic standards of that period, while from 2013 onward the European LeukemiaNet (ELN) 2013 definitions were used.
• Line of therapy*:* each distinct interval during which a patient receives a specific TKI before switching to another
• TFR-eligible refers to patients who fulfilled established guideline criteria for treatment-free remission: at least 5 years of uninterrupted TKI therapy, with ≥2 consecutive years of sustained deep molecular response (MR4 or deeper), and no prior history of TKI resistance, while remaining under active follow-up.
• TFR-achieved, by contrast, refers to patients in whom therapy was actually discontinued under physician supervision and who maintained treatment-free remission according to monitoring criteria.

### Statistical and predictive modeling

Descriptive statistics were used to summarize patient demographics and treatment patterns. To identify patients with the potential for successful TFR, we developed and compared two distinct machine learning models (Model A and Model B) using Random Forest, Gradient Boosting, and Logistic Regression algorithms. Model A included all engineered patient features for maximum predictive accuracy, whereas Model B excluded six endpoint features (e.g., min_bcr_abl_ever, achieved_mr4_ever) to simulate a real-world scenario where such information may not be available early in the treatment course. Models were evaluated on accuracy, sensitivity, and specificity, with performance validated using stratified 5-fold cross-validation.

## Results

Baseline characteristics are shown in Table 1.

**TABLE 1:** Baseline age and sex distribution.

Age bracket	F	M	All
0–18	0	0	0
18–25	3	4	7
25–35	3	10	13
35–45	12	9	21
45–55	16	28	44
55–65	11	20	31
65–75	31	18	49
75–85	14	17	31
85+	3	2	5
All	93	108	201

Regarding age Distribution at TKI Initiation, Imatinib (median 53, mean 50.7), dasatinib (50, 49.7), and nilotinib (52, 49.0) show comparable starting ages, while later-line agents trend older (bosutinib 56/54.7; ponatinib 50/50.5; asciminib 67/63.0).

Most patients (94.5%) presented in chronic phase; 5.5% in accelerated phase, as shown in Table 2.

**TABLE 2:** Phase of disease.

Phase	Number of cases
Chronic	190
Accelerated	11
Total	201

Baseline prognostic scores such as SOKAL or EUTOS could not be assessed, as the necessary laboratory data were not available consistently across the cohort.

### Medication per line of therapy

The distribution of TKI use across different lines of therapy is summarized below. Imatinib was the most common first-line agent, with dasatinib and nilotinib dominating second-line therapy. Bosutinib, ponatinib, and asciminib were used primarily in third-line and later settings (Supplementary Figures S1A–F).

The reasons for discontinuing each TKI were analyzed (Table 3), adverse reaction analysis (Table 4), including achievement of TFR, TFR eligibility, adverse effects, and loss of response.

**TABLE 3:** Medication discontinuation summary.

Medication	Asciminib (*n* = 11)	Bosutinib (*n* = 13)	Dasatinib (105)	Imatinib (105)	Nilotinib (*n* = 68)	Ponatinib (*n* = 11)	Total
TFR	0	0	3	3	8	0	14
Continuing treatment, TFR eligible	0	0	9	16	6	0	31
Adverse events	2	6	23	17	9	4	61
Loss of therapeutic response	1	1	8	34	9	1	54
Continuing treatment, TFR not eligible	8	6	62	35	36	6	153

**TABLE 4:** Adverse reactions analysis.

	Medication	Asciminib (*n* = 11)	Bosutinib (*n* = 13)	Dasatinib (*n* = 105)	Imatinib (*n* = 105)	Nilotinib (*n* = 68)	Ponatinib (*n* = 11)	Total
Category	Subcategory							
Hematologic	Grade ≤3	1	0	4	2	4	0	11
	Grade 4	0	1	1	0	1	1	4
Non-hematologic	Cardio-circulatory	0	0	2	0	1	0	3
	Pulmonary (Pleurisy)	0	2	10	0	0	0	12
	Dermatologic	0	1	2	1	1	2	7
	Digestive	0	1	2	2	0	0	5
	Unspecific	1	1	2	12	2	1	19
Total		2	6	23	17	9	4	61

Across TKIs, AE-related discontinuations varied substantially: 16.2% for imatinib (17/105), 21.9% for dasatinib (23/105), 13.2% for nilotinib (9/68), 46.2% for bosutinib (6/13), 36.4% for ponatinib (4/11), and 18.2% for asciminib (2/11). These data highlight the differences in tolerability across agents and are presented here in the main text for accessibility, while detailed AE types and distributions remain available in the Supplementary Figures.

Table 5 shows the top 10 correlated features with TFR potential.

**TABLE 5:** Top 10 correlated features with TFR potential.

Feature	Correlation	p_value	Significant
achieved_mr4_ever	0.38988	0.00000	True
total_drug_switches	−0.20557	0.00342	True
latest_bcr_abl	−0.19599	0.01066	True
imatinib_duration	0.17446	0.01325	True
early_vs._late_improvement	0.15309	0.21976	False
early_response_speed	0.14932	0.21730	False
nilotinib_duration	0.14247	0.04364	True
best_response_after_year1	−0.13552	0.09487	False
min_bcr_abl_ever	−0.13313	0.08444	False
disease_phase_chronic	0.13108	0.06363	False
had_adverse_events	−0.13092	0.06395	False
time_to_mr4_months	−0.12294	0.17730	False
eo_count_baseline	−0.12255	0.11244	False
hgb_baseline	0.11667	0.13087	False
bcr_abl_trend_slope	−0.11120	0.19747	False

To understand the potential for Treatment-Free Remission (TFR) in patients, a comprehensive analysis of various patient features was conducted. The table above, “Top 20 Correlated Features with TFR Potential,” summarizes the most impactful factors identified through this analysis, highlighting their correlation with TFR potential, their statistical significance (p-value), and whether they meet the threshold for significance. Note: Imatinib’s substantial impact on cohort outcomes reflects its early and prolonged use as the first widely available TKI in Romania.

### Strong positive correlations and their implications

• achieved_mr4_ever (0.38988, p = 0.00000, True): This feature exhibits the strongest positive correlation with TFR potential, indicating that achieving a Major Molecular Response 4 (MR4) at any point during treatment is a highly significant predictor of successful TFR. This underscores the importance of deep molecular responses in the therapeutic journey of these patients.
• imatinib_duration (0.17446, p = 0.01325, True): A longer duration of imatinib treatment is positively correlated with TFR potential. This suggests that sustained exposure to the initial tyrosine kinase inhibitor (TKI) can contribute to a more stable disease control, potentially priming patients for a successful cessation of therapy.
• nilotinib_duration (0.14247, p = 0.04364, True): Similarly, a longer duration of nilotinib treatment, another TKI, also shows a positive and significant correlation with TFR potential. This reinforces the idea that prolonged exposure to effective TKI therapy, regardless of the specific agent, can positively influence the likelihood of TFR.

### Significant negative correlations and their insights

• total_drug_switches (-0.20557, p = 0.00342, True): This feature demonstrates a significant negative correlation, implying that a higher number of drug switches during treatment is associated with a lower likelihood of achieving TFR. This could be due to disease resistance, intolerance to previous treatments, or a more complex disease course, all of which might hinder successful treatment discontinuation.
• latest_bcr_abl (-0.19599, p = 0.01066, True): A higher (less favorable) latest BCR-ABL transcript level is negatively correlated with TFR potential. This highlights that consistently low levels of the BCR-ABL transcript, a key marker of disease, are crucial for considering TFR. Patients with higher residual disease burden are less likely to maintain remission off therapy.

**FIGURE 1:**
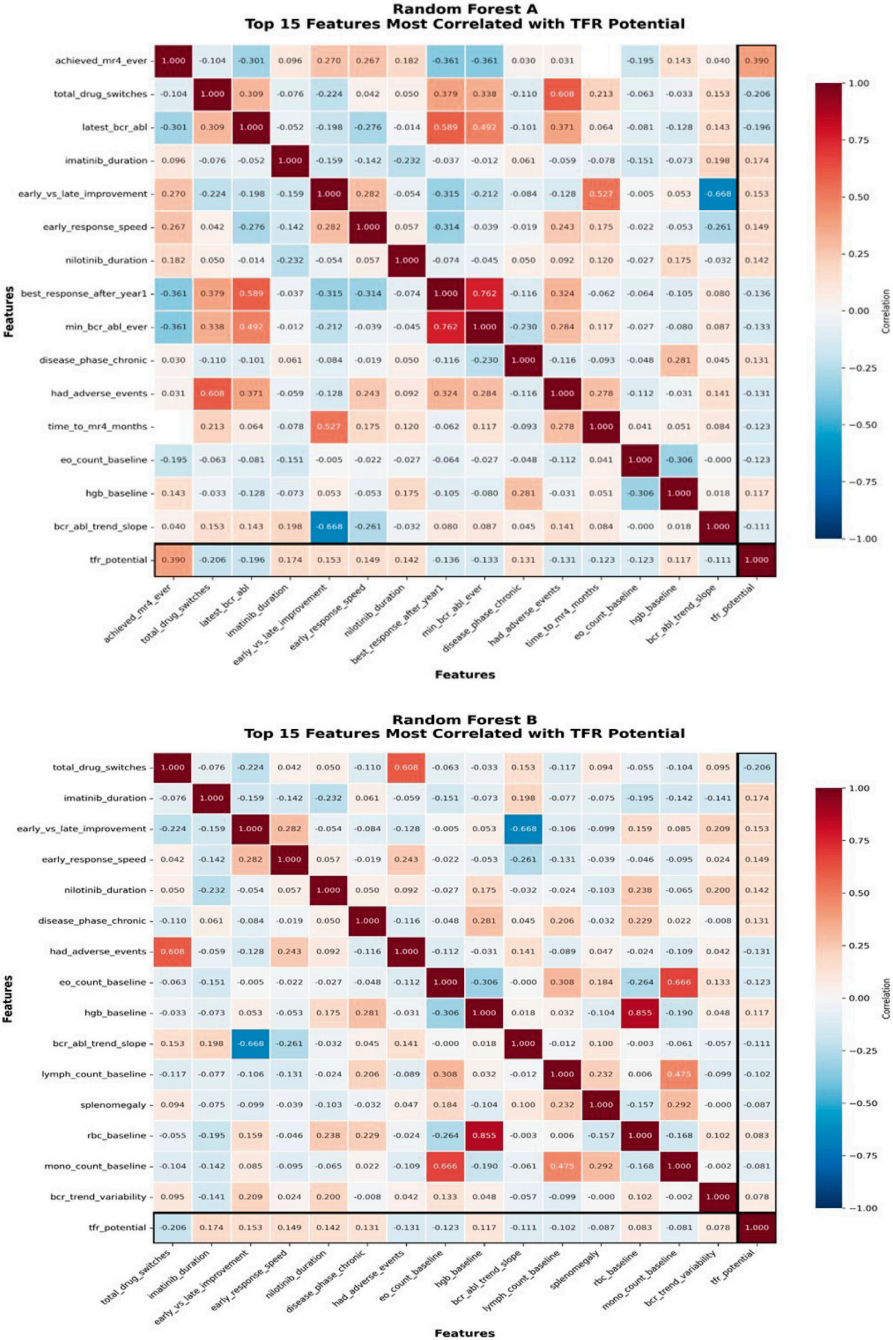
Random Forest **(A)**. Top 15 features most correlated with TFR potential. Random Forest **(B)**. Top 15 features most correlated with TFR potential.

Figures 1A,B depicts heatmaps of the best the top 15 features of each mode.

### Predictive modeling of treatment-free remission potential

Among the models trained to predict TFR potential, the Model A Random Forest classifier achieved the highest performance, with an accuracy of 85.4%, a sensitivity of 77.8%, and a specificity of 87.5%. When endpoint-defining features were excluded for earlier predictions in Model B, the Random Forest classifier remained the top performer, yielding an accuracy of 82.9%, sensitivity of 44.4%, and specificity of 93.8%.

Analysis of the top-performing Model A revealed that the most important features for predicting TFR potential were latest_bcr_abl (most recent BCR-ABL measurement), best_response_after_year1 (lowest BCR-ABL after the first year), min_bcr_abl_ever (deepest response ever), time_to_mr4_months (time to reach MR4), and achieved_mr4_ever (binary flag for ever reaching MR4). This finding was consistent with statistical analysis, where achieved_mr4_ever showed the strongest correlation with TFR potential (r = 0.39, p < 0.001) (Table 6).

**TABLE 6:** Statistical analysis models, where achieved_mr4_ever showed the strongest correlation with TFR potential.

Model	Accuracy	F1	Sensitivity	Specificity
Random Forest A	0.854	0.802	0.778	0.875
Gradient Boosting A	0.854	0.767	0.556	0.938
Random Forest B	0.829	0.714	0.444	0.938
Gradient Boosting B	0.683	0.470	0.111	0.844
Logistic Regression A	0.610	0.566	0.667	0.594
Logistic Regression B	0.610	0.549	0.556	0.625

Feature importance analysis revealed that molecular response kinetics and endpoints were the most significant predictors. For the top-performing Random Forest A model, the most predictive features were the latest available BCR-ABL value (latest_bcr_abl), the best response achieved after the first year of treatment (best_response_after_year1), and the minimum BCR-ABL level ever recorded (min_bcr_abl_ever). Other key predictive features included whether a patient ever achieved MR4.5 (achieved_mr4_ever), the consistency of their response (response_consistency_r2), and the variability of their BCR-ABL trend (bcr_trend_variability).

## Discussions

Imatinib remains the therapeutic backbone in our setting, accounting for 53.7% of first-line courses—and thus defines baseline expectations for efficacy and tolerance. Its continued predominance is partly explained by the fact that many patients in our cohort-initiated treatment during a period when imatinib was the only available TKI option in Romania. Over time, a substantial proportion of these patients have achieved durable deep molecular responses, reflecting the drug’s long-term effectiveness in real-world practice. In our cohort, MR4+ was documented in 29% of imatinib exposures, contributing to 3 of the 14 observed TFR successes and 16 of the 31 TFR-eligible cases (51.6%). While this shows that imatinib can achieve durable deep responses, the conversion rate from eligibility to successful TFR is modest when compared with nilotinib, emphasising the importance of structured stop protocols and enhanced molecular monitoring in patients who remain on imatinib for many years. Adverse events led to discontinuation in 16.2% of imatinib-treated patients (17/105), mainly low-grade, while loss of therapeutic response accounted for 32.4% of discontinuations (34/105), highlighting the need for timely molecular monitoring and consideration of switch strategies when treatment milestones are missed.

Dasatinib demonstrated the highest on-treatment persistence in the study, with 59% of patients (62/105) remaining on therapy. Despite a pleural effusion rate of 10% (10/105), adverse events led to discontinuation in 21.9% of dasatinib-treated patients (23/105). Dasatinib produced three TFR successes and nine additional TFR-eligible patients, confirming its ability to induce sustained MR4 when introduced either front-line (27.9% of 1L starts) or as early salvage (64.1% of 2L switches). Nilotinib emerged as the cohort’s most effective TFR-enabler. It accounted for eight of the fourteen TFR achievements (57.1%) and six additional TFR-eligible cases, reflecting strong performance in achieving deep molecular responses. Cardiovascular toxicity, historically a concern, was limited to a single grade 2 arterial event (1.5%), indicating improved safety in a real-world setting. Nilotinib also demonstrated robust salvage efficacy, delivering MR4+ in 43.3% of third-line administrations.

Later-line agents played narrower roles. Bosutinib was mainly used in third-line or beyond, with the highest AE-related discontinuation rate (46.2%, 6/13). At the same time, 46.2% of patients (6/13) remained on therapy at analysis, reflecting heterogeneity in treatment persistence. Ponatinib retained value as a mutation-directed option, particularly for T315I, with no arterial events observed, though median exposure was short. Asciminib, introduced only from third line onward, showed manageable toxicity and, although no patients yet reached TFR eligibility, its mutation-agnostic profile and cardiovascular safety may expand future candidacy.

Regarding the cross-agent themes and Machine Learning Insights, the second-generation TKIs now represent 46.3% of first-line therapy, aligning local practice with 2024 NCCN and ELN guidance favouring deeper molecular responses. (ii) Adverse-event management—especially pleural effusion on dasatinib and metabolic/cardiovascular monitoring on nilotinib—directly influences continuation rates. (iii) The gap between TFR eligibility (15.4%) and actual TFR (7.0%) highlights the operational need for standardized discontinuation pathways and high-sensitivity BCR-ABL1 PCR (MR4.5) availability. Our findings also align with, and diverge from, large registries and landmark trials. In our cohort, imatinib remained the predominant first-line TKI (53.7%), whereas SEER registry data show increasing use of second-generation TKIs in more recent years [3,4]. The observed TFR achievement rate of 7% was lower than the 40%–55% reported in prospective trials such as ENEST (nilotinib vs. imatinib) and DASISION (dasatinib vs. imatinib) [13,14], reflecting differences in monitoring intensity, access to high-sensitivity PCR, and availability of structured stop protocols. More recently, the ASC4FIRST trial demonstrated superior molecular response rates with asciminib compared with physician’s choice of TKI in the frontline setting [8], underscoring the evolving therapeutic landscape.

Our predictive modeling analysis reinforces the clinical observations but also emphasizes important methodological aspects. Model A achieved the highest accuracy (85.4%), but this performance was largely driven by endpoint-defining features such as ever achieving MR4.5. Although these variables are well-recognized biological markers of TFR potential, their inclusion introduces a risk of circularity, meaning that the model’s accuracy may overestimate its practical predictive value. Therefore, the utility of Model A is mainly retrospective, identifying which features distinguish patients once their treatment course has already unfolded. This model can only provide prospective value after the patient has already achieved MR4.5, but the 2 years required for TFR clinical guidelines have not yet passed. Model B attempted to address the issue of circularity and provide earlier prospective applicability by excluding endpoint-defining features. While accuracy remained acceptable (82.9%), sensitivity decreased to 44.4%, which limits its immediate clinical usability. This discrepancy highlights the difficulty of predicting TFR eligibility with early longitudinal data, particularly when standardized molecular monitoring intervals are not consistently available. The models presented here should be regarded as prototypes that demonstrate the feasibility of ML-based TFR prediction. While they achieved encouraging performance (85.4%) within our cohort, clinical application would necessitate training on larger, multi-center datasets, further refinement and validation in external cohorts. This indicates that machine learning could ultimately become a useful tool to support clinical decision-making for CML patients being evaluated for treatment cessation, with the potential to identify suitable candidates earlier in their therapeutic course.

Finally, the gap between TFR eligibility (15.4%) and achievement (7%) reflects system-level barriers. Limited access to high-sensitivity PCR, variability in physician practice, and absence of standardized stop-protocols likely contributed to underutilization. Addressing these barriers, alongside advances in predictive modeling, may expand safe adoption of TFR in routine practice.

This study has several limitations. Its retrospective design and single-centre scope may limit the generalizability of the findings. The availability of molecular data was dependent on the timing of routine clinical visits rather than strictly defined intervals (e.g., 30, 90, 180 days), which may affect the precision of kinetic analyses. Additionally, comprehensive kinase domain mutation profiling was not available for all patients who experienced treatment failure. Phase classification followed physician-recorded criteria at the time of care, with ELN 2013 definitions applied only in later years. This reflects real-world practice but introduces some heterogeneity across the 24-year study period. Regarding treatment, the predominance of imatinib in this cohort reflects its historical status as the only available TKI in Romania, which may influence long-term outcome patterns and limit generalisability to settings with earlier access to second-generation TKIs. Furthermore, the 24-year observation period encompasses major changes in treatment guidelines, monitoring standards, and assay sensitivity, introducing temporal heterogeneity that may bias longitudinal comparisons and must be considered when interpreting the results.

The use of a Large Language Model for data extraction, while innovative and efficient, is dependent on the model’s accuracy, and the possibility of extraction errors cannot be entirely eliminated despite cleaning and validation steps. Finally, while the predictive Model A demonstrated high accuracy, it included features that are endpoint-defining, creating a risk of feature tautology. Model B was designed to mitigate this, but at the cost of lower predictive power, highlighting the trade-off between accuracy and early clinical applicability.

## Conclusions and implications in clinical practice

In the frontline landscape, imatinib still dominates, reflecting its historical status as the sole available option in Romania and its durable efficacy in long-term patients, but a combined 46.3% of first-line starts now use dasatinib or nilotinib, aligning with 2024 NCCN preference for 2G TKIs in high-risk disease. Optimal switch strategy includes the early molecular failure, that should prompt a switch from imatinib to nilotinib, which demonstrated favorable continuation and deep response rates. Regarding efficacy, nilotinib is the cohort’s most effective TFR-enabler, with (11.7% vs. 4.4% overall) TFR percentage of treatment outcomes, as well as 20.5% vs. 14.4% overall) TFR + TFR-eligible percentage of treatment outcomes. Structured stop-protocols could raise current TFR attempts (7%–22.4%) of patients.

As future directions, routine BCR-ABL1 kinase-domain sequencing, cardiovascular risk stratification, and high-sensitivity PCR (MR4.5) will be crucial for precision sequencing and expanding treatment-free remission in Romanian practice.

## Data Availability

The original contributions presented in the study are included in the article/Supplementary Material, further inquiries can be directed to the corresponding authors.
