# Supplementary figures and images for: In-depth analysis for TKI-driven real-world management of 201 CML patients using TFR
Source: Front Pharmacol. 2025 Nov 6;16:1673056. doi: 10.3389/fphar.2025.1673056 (PMC12631139; doi:10.3389/fphar.2025.1673056)

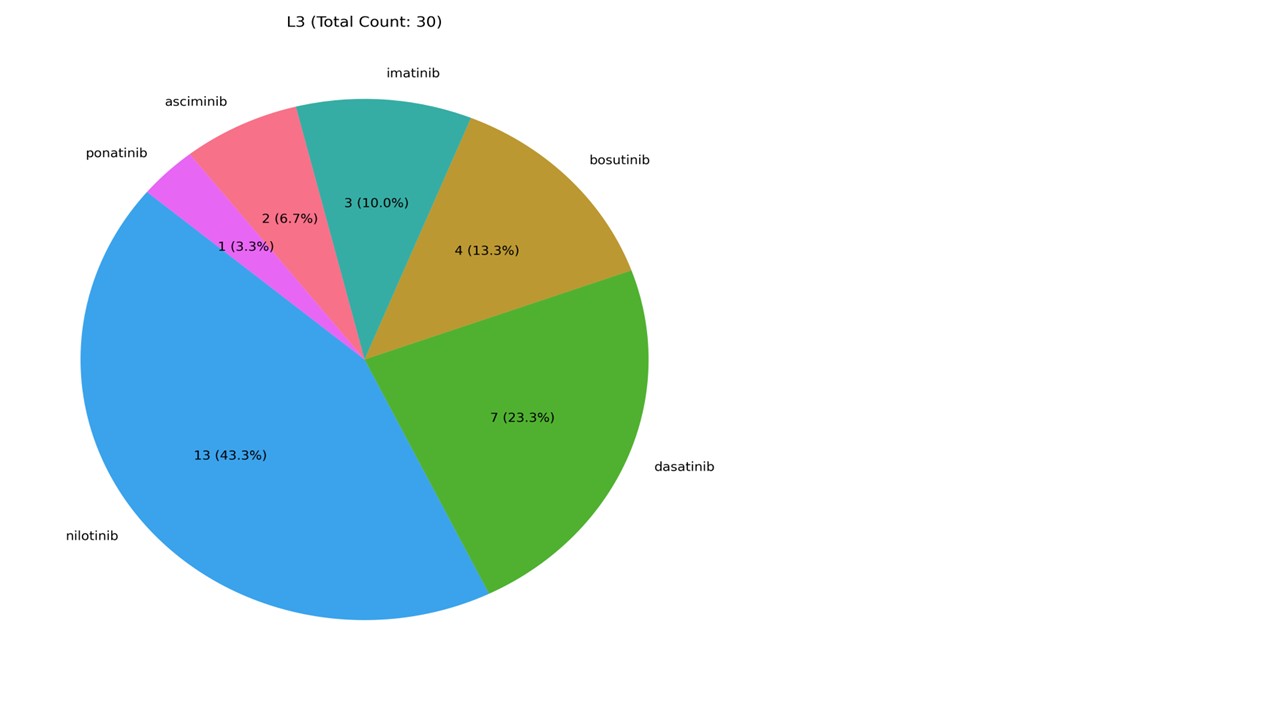

Supplement: Supplementary file 1 [file Image3.jpeg]

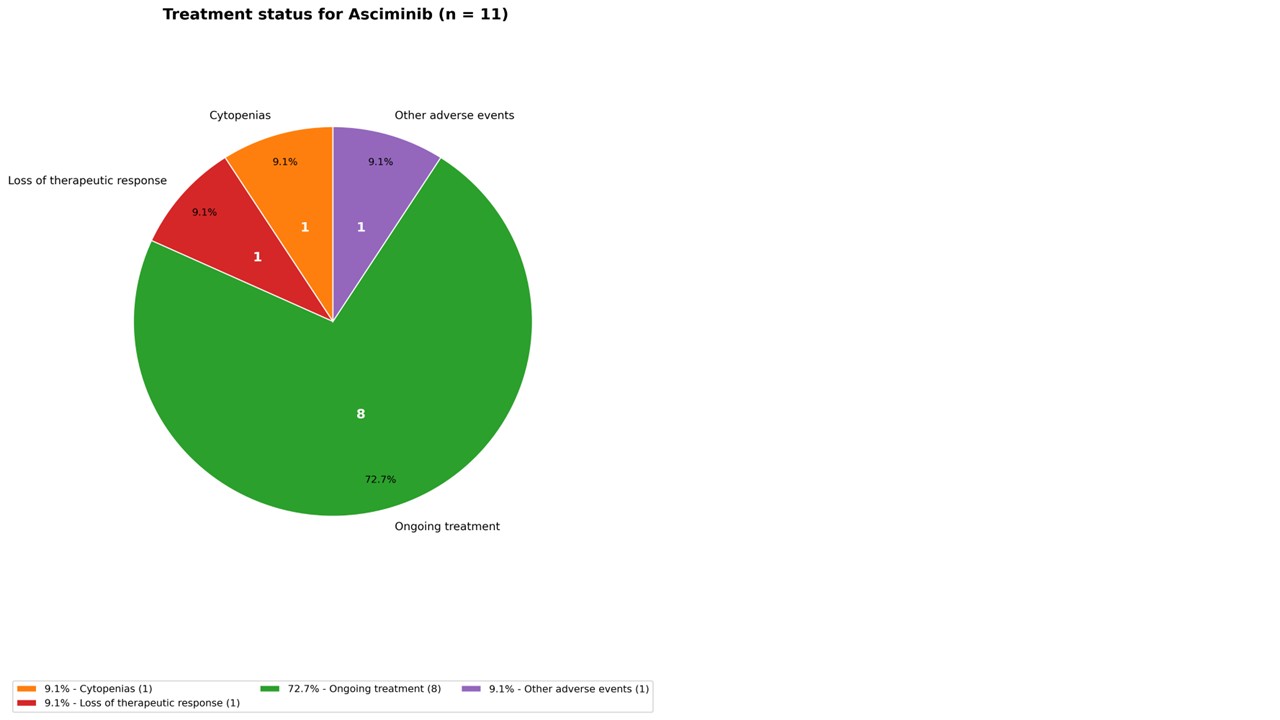

Supplement: Supplementary file 2 [file Image9.jpeg]

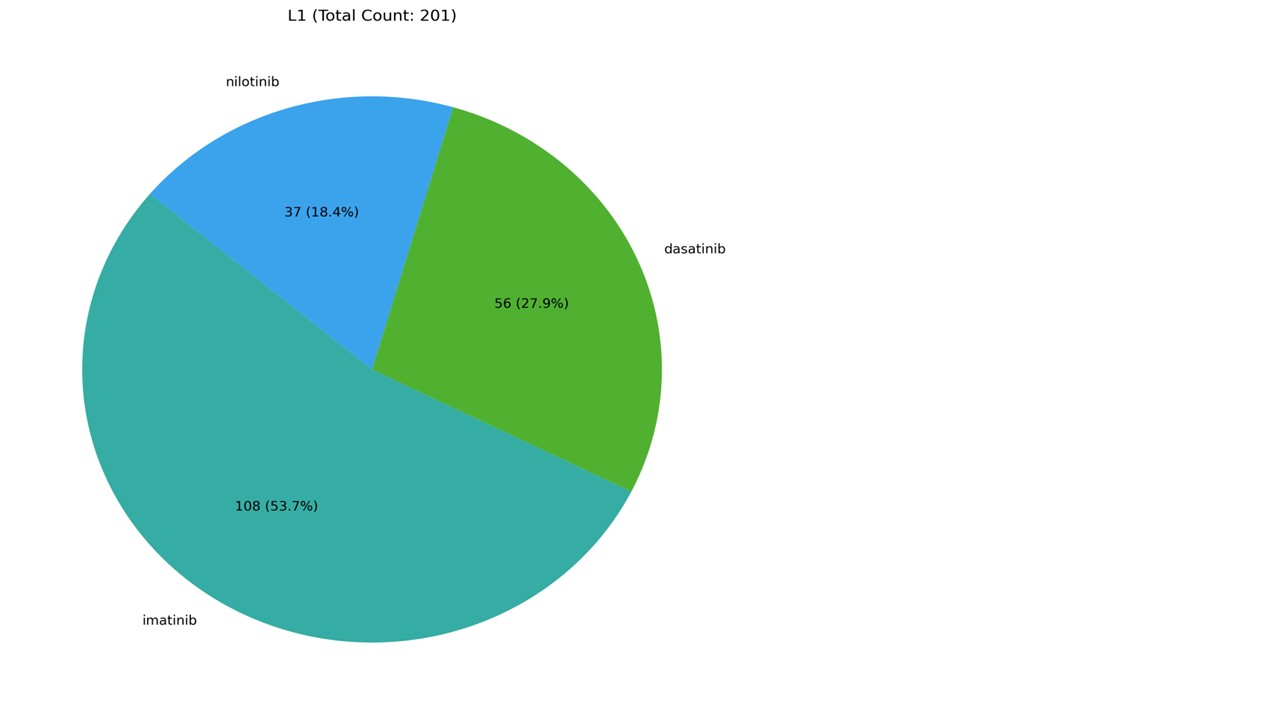

Supplement: Supplementary file 3 [file Image1.jpeg]

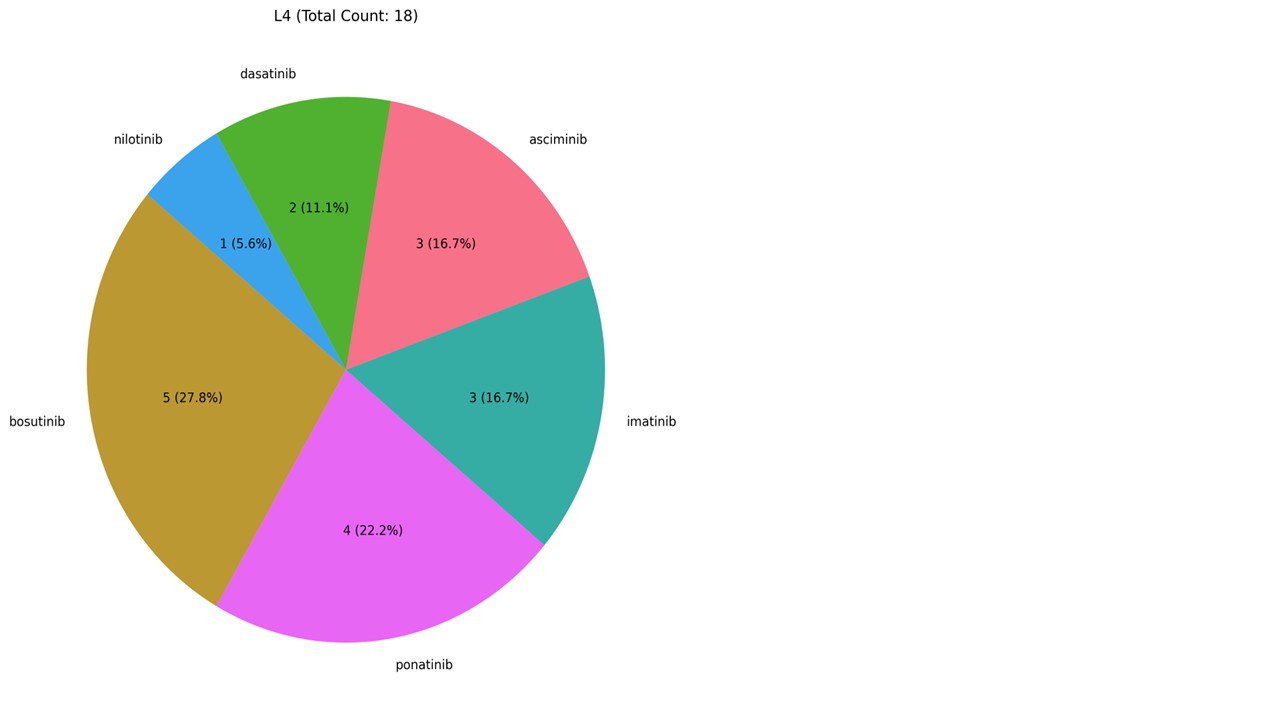

Supplement: Supplementary file 4 [file Image4.jpeg]

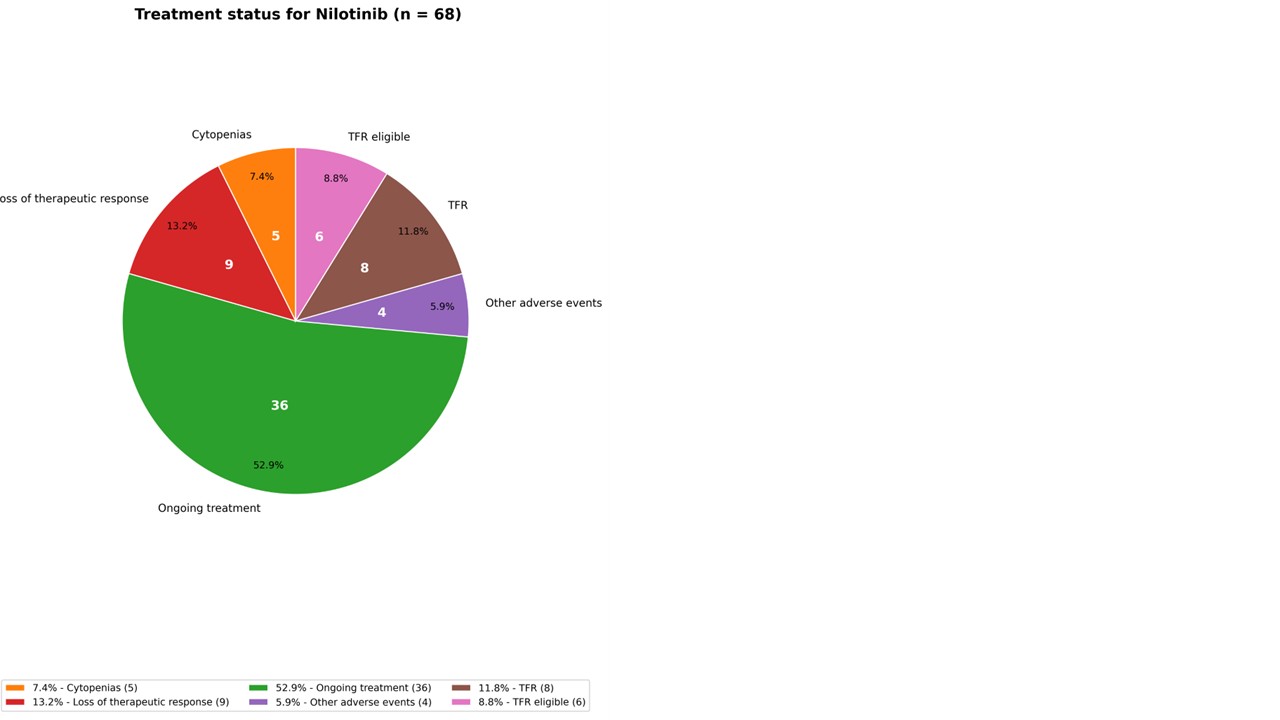

Supplement: Supplementary file 5 [file Image7.jpeg]

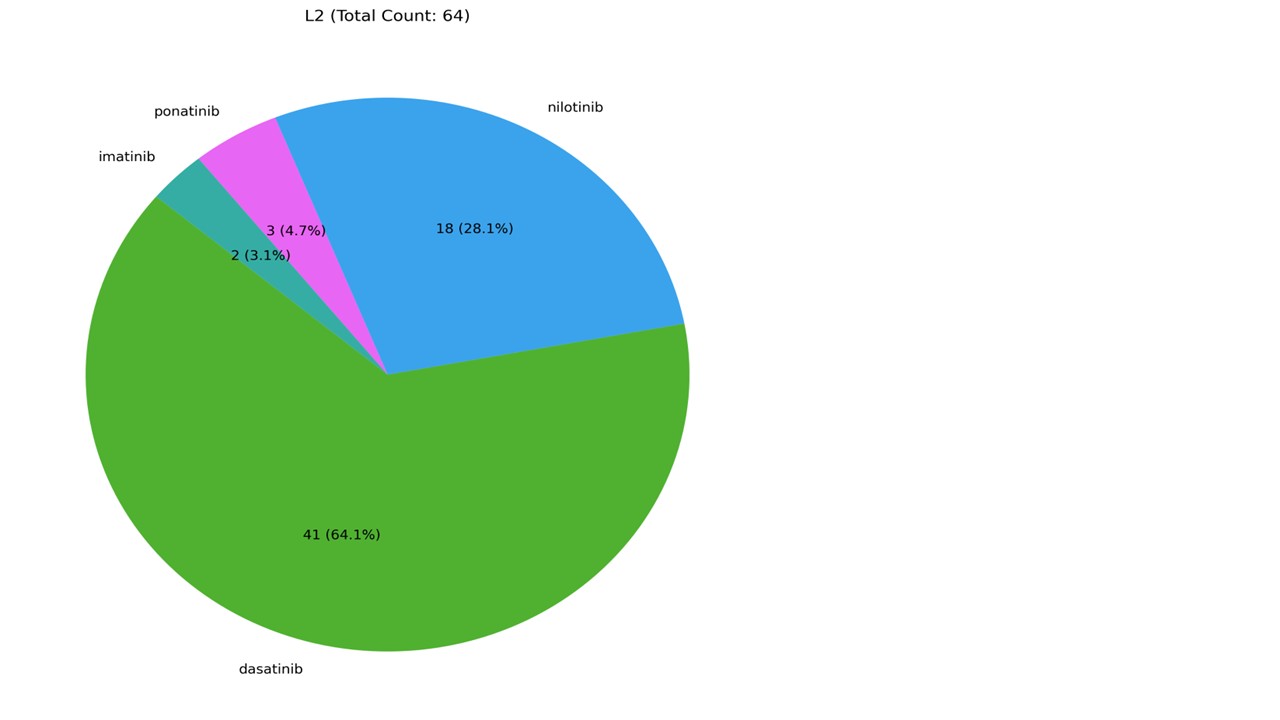

Supplement: Supplementary file 6 [file Image2.jpeg]

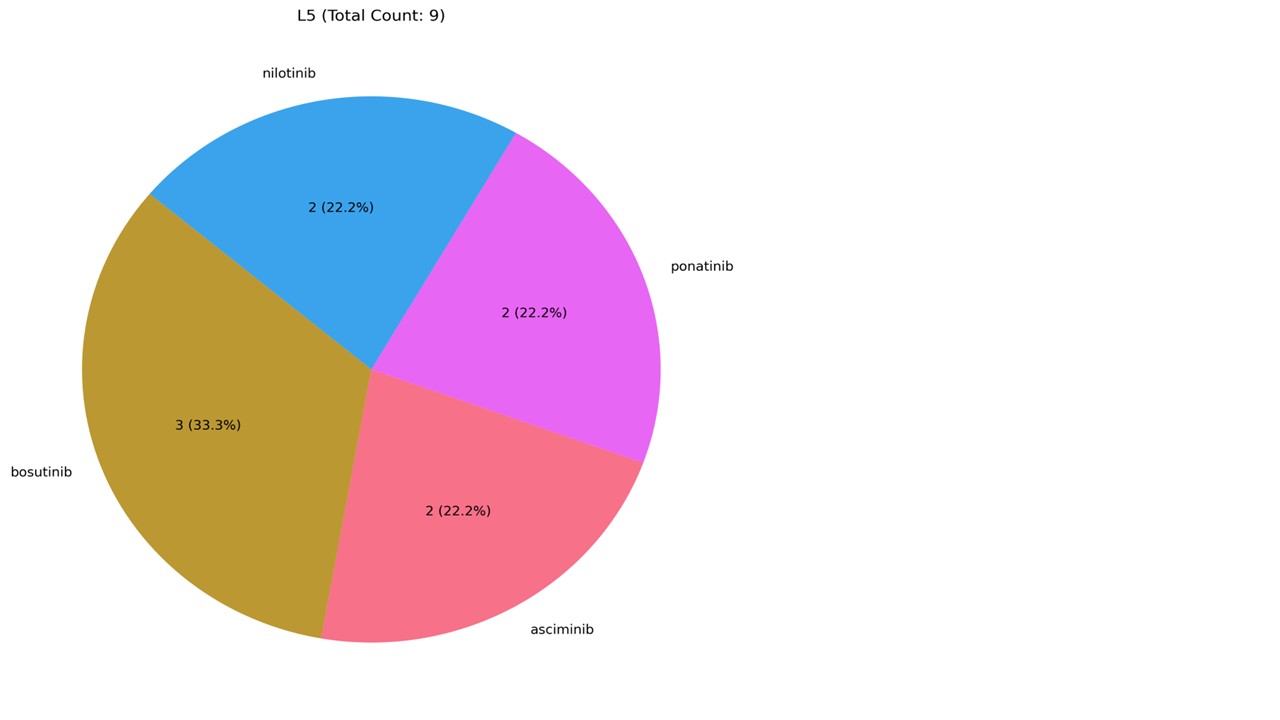

Supplement: Supplementary file 7 [file Image5.jpeg]

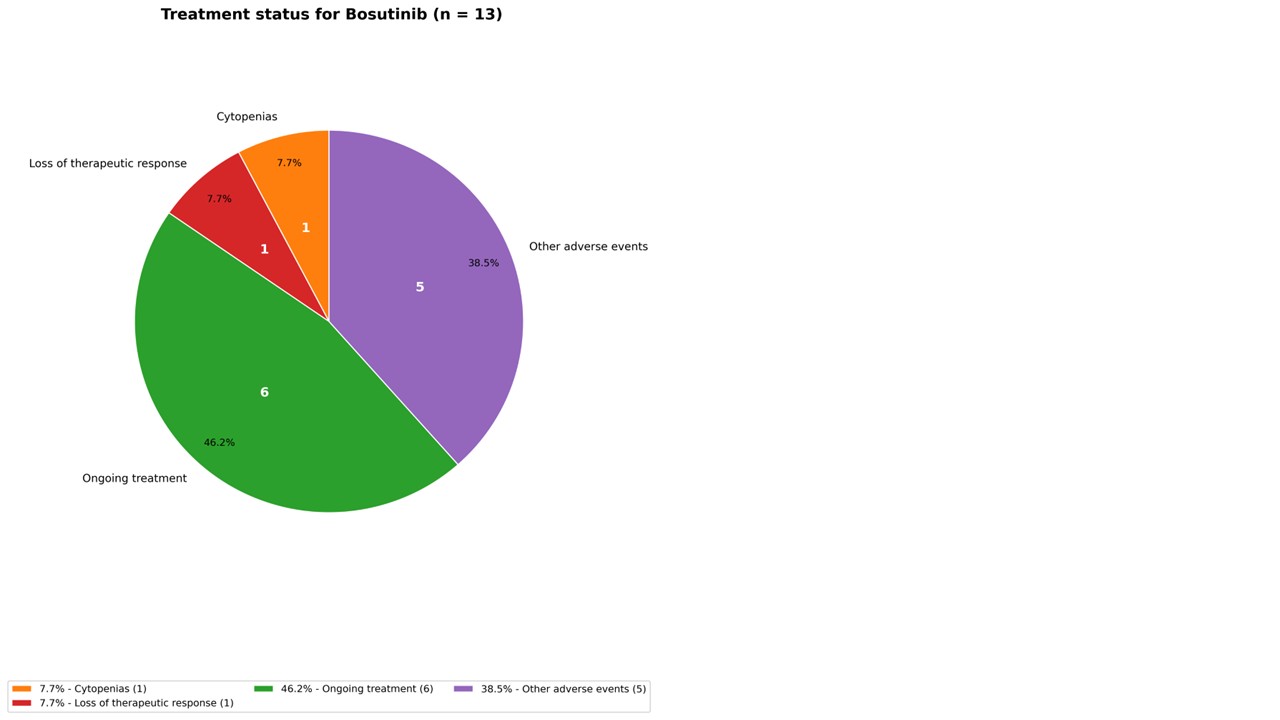

Supplement: Supplementary file 8 [file Image10.jpeg]

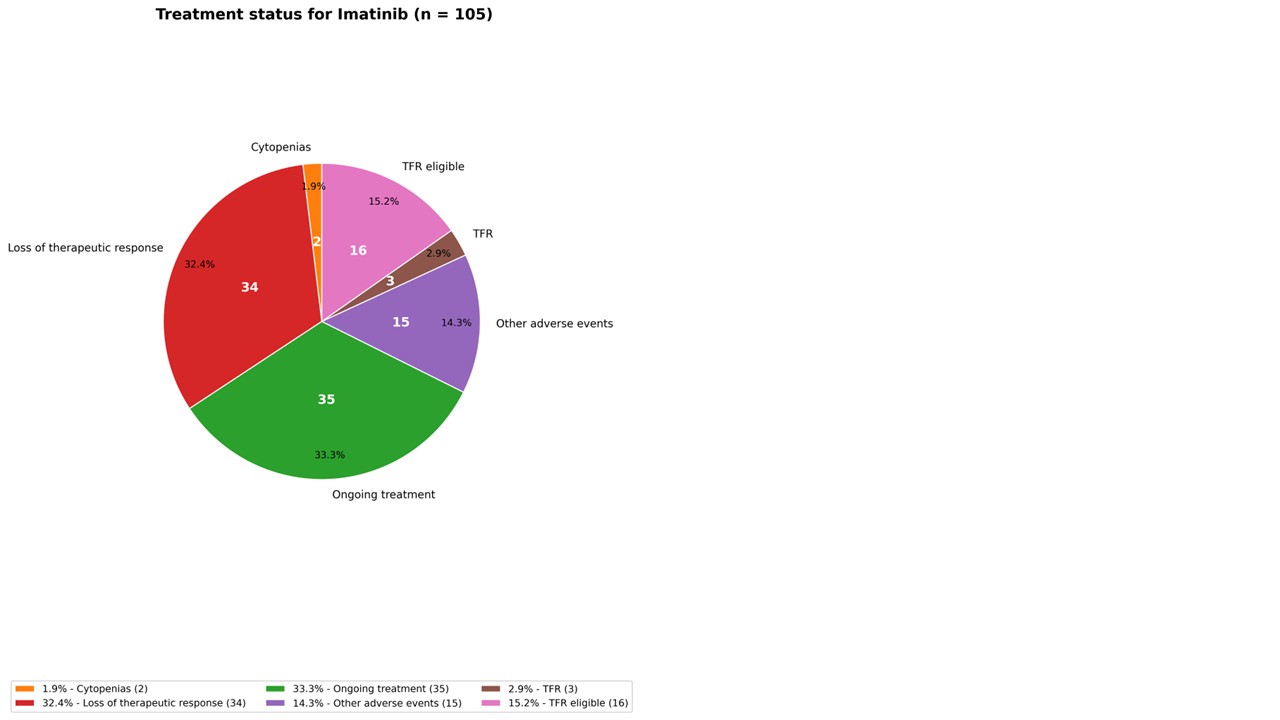

Supplement: Supplementary file 9 [file Image12.jpeg]

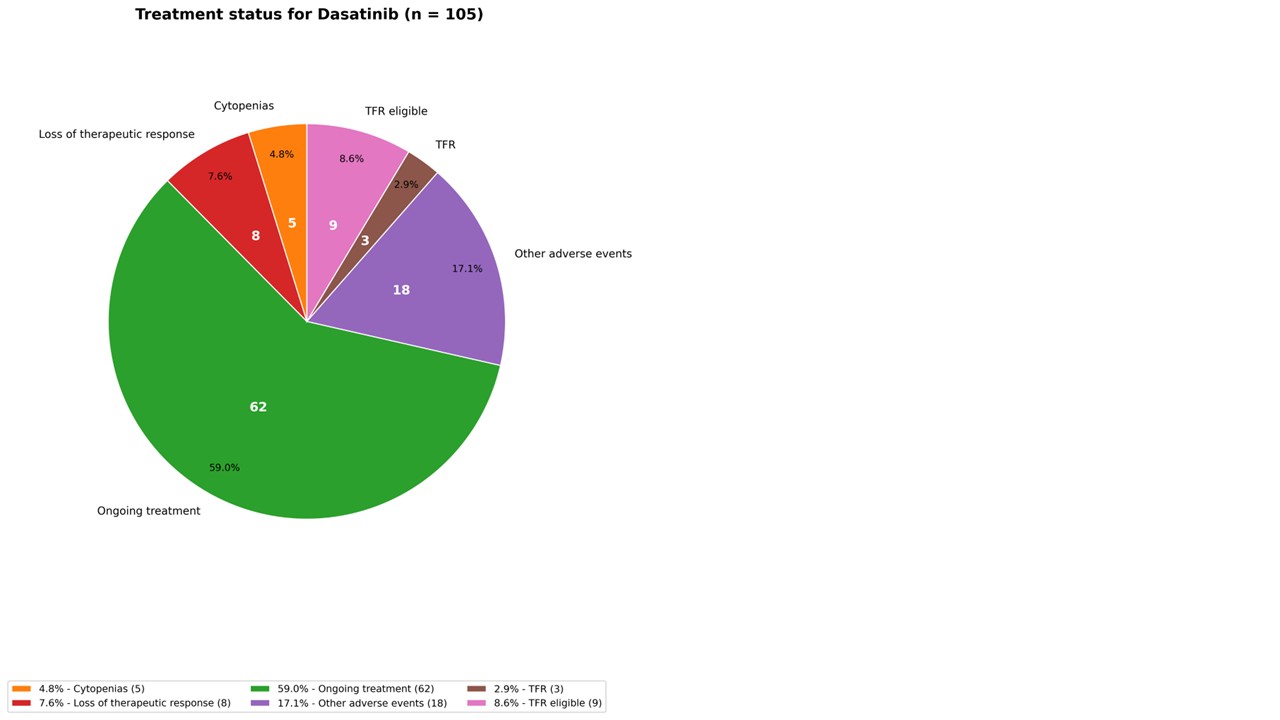

Supplement: Supplementary file 10 [file Image11.jpeg]

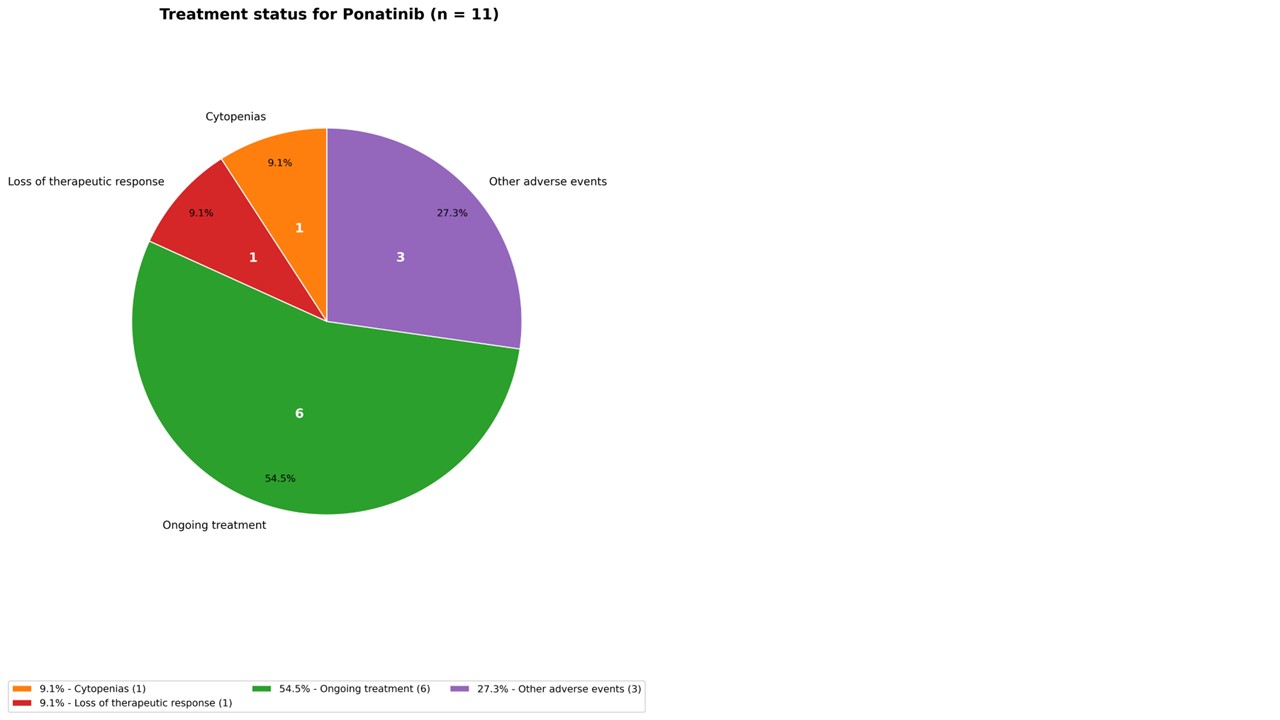

Supplement: Supplementary file 11 [file Image8.jpeg]

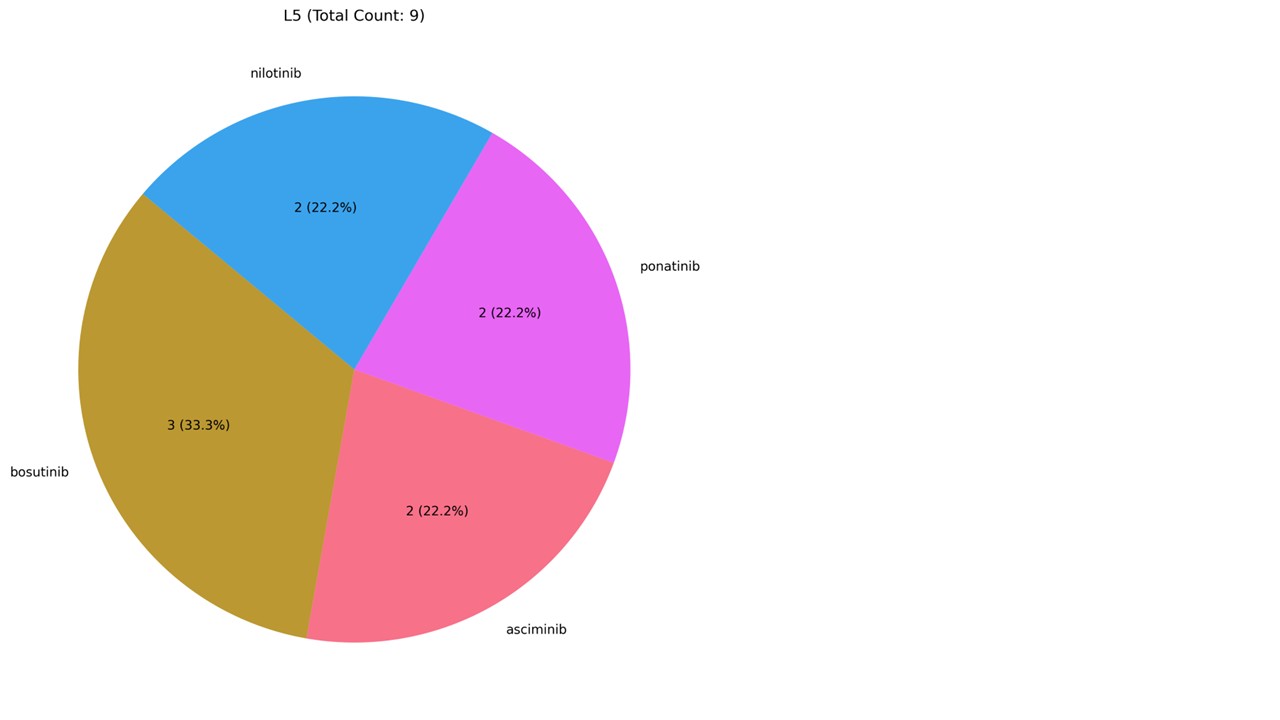

Supplement: Supplementary file 12 [file Image6.jpeg]
